# Band 40/41 Surface Acoustic Wave Filters on 42°YX-Lithium Tantalate Substrate with Suppression of Transverse Leakage

**DOI:** 10.3390/mi15050585

**Published:** 2024-04-28

**Authors:** Qiang Xiao, Zihang Gao, Mengqun Chu, Zeyu Zheng, Xuesong Du, Chengji Hu, Hongzhi Pan, Hualin Li, Jiahe Dong, Zhenglin Chen, Huazhi Chen, Chuan Lu, Mi Tang, Yanping Fan, Jinyi Ma

**Affiliations:** 1 China Electronics Technology Group Corp 26th Research Institute, Chongqing 400060, China; chumengqun@foxmail.com (M.C.); 13752882051@163.com (Z.Z.); duxs@cetccq.com.cn (X.D.); hucj@cetccq.com.cn (C.H.); plotinpan95@yeah.net (H.P.); dongjh@cetccq.com.cn (J.D.); zlchen1988@yeah.net (Z.C.); cetc26chz@126.com (H.C.); weeeee1984@163.com (C.L.); lk13896012970@163.com (M.T.); 2 School of Electronic Information and Electrical Engineering, Shanghai Jiao Tong University, Shanghai 200240, China; shjdgzh26108@sjtu.edu.cn (Z.G.); lihl@cetccq.com.cn (H.L.); 3 School of Optical-Electrical and Computer Engineering, University of Shanghai for Science and Technology, Shanghai 200240, China; fypofcas@163.com

**Keywords:** band 40/41, SAW filter, LiTaO_3_ substrate, transverse leakage, wider dummy

## Abstract

The transverse leakage of leaky surface acoustic waves (LSAWs) occurs on 42°YX-lithium tantalate substrates (42LT), which increases the insertion loss, narrows the bandwidth and flattens the roll-off of band 40/41 SAW filters and duplexers. In this work, LSAW characteristics with different metal materials and thicknesses are calculated by the finite element method (FEM), which determines the IDT material and thickness used for band 40/41 SAW filter design. To deeply understand transverse leakage and suppress it, the effects of different gap and dummy lengths on transverse leakage are simulated and discussed. Then, a new technique of using a wider dummy without any additional lithographic or depositing processes is proposed to suppress the leakage. Its effectiveness is validated by both simulations and experiments. Then, the technique is extended to applications of band 40 and 41 SAW filters. The experimental results show that with the wider dummy structure, the band 40 and 41 SAW filters achieve a more than 0.2 dB improvement in the insertion loss, a wider bandwidth and a steeper roll-off characteristic. This technique may also be extended to the other band SAW filter applications.

## 1. Introduction

Bands 40/41 are important bands for the CHINA MOBILE 4G communication of TD-SCDMA’s long-term evolution (TD-LTE) [1]. Presently, surface acoustic wave (SAW) filters and duplexers are the main solution for bands 40 (2320 MHz–2370 MHz) and 41 (2515 MHz–2675 MHz) due to their small size, low cost and steady performance [2].

A leaky SAW (LSAW) on a 42°YX-lithium tantalate (LiTaO_3_, LT) substrate is a competitive candidate for band 40/41 SAW filters and duplexers thanks to its high SAW velocity and release from spurious transverse mode generation [3,4]. However, a LSAW on 42°YX-LiTaO_3_ transversely penetrates its energy into the busbar region [5,6], especially in high-frequency applications. This transverse leakage effect will deteriorate the performance of SAW filters and duplexers, e.g., by increasing the insertion loss, narrowing the bandwidth or flattening the roll-off.

The transverse effects have been actively studied for a long time. The phenomenon of transverse leakage was discovered and investigated by Koskela et al. [4] using laser interferometric measurements. They concluded that the leakage was caused by the velocities’ differences and strongly depended on frequency. A generalized 3D FEM/BEM model was introduced by Solal et al. [7] to analyze and predict the effects of a resonator, including an electrode end gap and dummy electrodes, but they did not propose any solution to suppress the transverse leakage. To suppress the unwanted effect, a narrow gap and long dummy are commonly employed [7]. But for high-frequency applications, transverse leakage still occurs. Tsutsumi and Inoue et al. [8,9] pointed out that transverse leakage could be suppressed by a smaller IDT metallization ratio (MR < 50%) to achieve ultra-low loss and steep roll-off characteristics, but this means a higher lithographic resolution for high-frequency devices. Then, Nakamura et al. [10] proposed a technique of depositing a Ta_2_O_5_ film on the outside of the IDT region to solve the same problem. This technique makes the LSAW velocity on the outside of the IDT region slower than that in the interdigital transducer (IDT) region to avoid transverse leakage. However, additional deposition is needed, which increases the manufacturing complexity and production cost. Solal et al. [11] proposed a new structure using the superposition of a standard resonator and a 2D grating of reflectors, similar to phononic crystals, to solve the same problem, but an additional tungsten layer was needed, and it was not entirely certain that the new device really worked as anticipated.

In this work, LSAW characteristics (*V_p_*, *K*^2^ and *TCF_r_*) with different metal materials and thicknesses are calculated by the finite element method (FEM) in Section 2. This determines the IDT material and thickness used for the band 40/41 SAW filter design in this work. To suppress transverse leakage, the effects of different gap and dummy lengths on transverse leakage are simulated and discussed, and a new technique without any additional lithographic or depositing processes is proposed in Section 3. Further, this technique is extended to the band 40 and 41 filter applications, and the measured results are presented and discussed in Section 4. Section 5 concludes the paper.

## 2. Simulation of LSAW Characteristics

**Figure 1:**
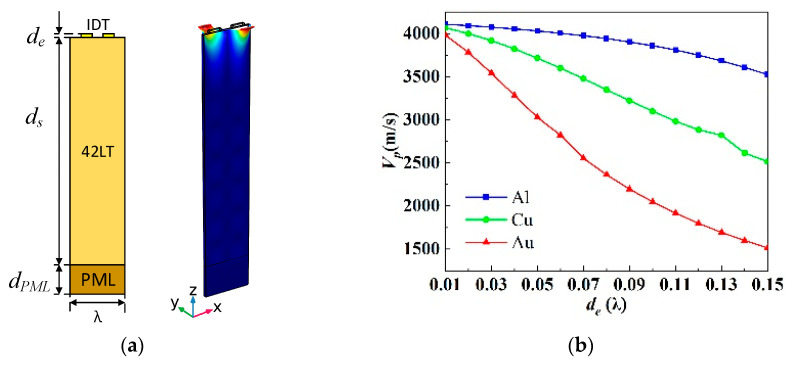

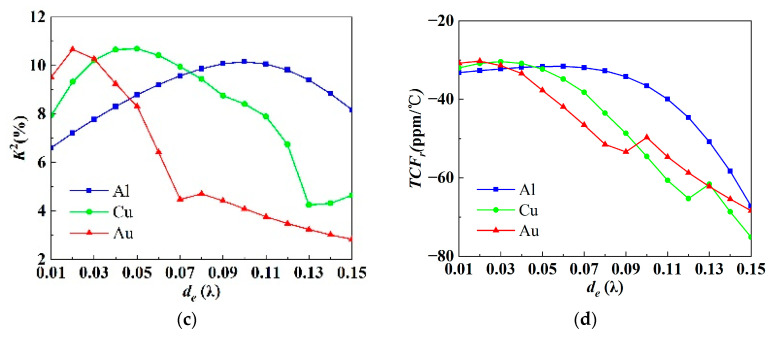
(**a**) Cross-sectional schematic of the IDT/42LT/PML structure with typical displacement of LSAW. (**b**) *V_p_*, (**c**) *K^2^* and (**d**) *TCF_r_* of the LSAWs as a function of *d_e_* with different metal materials (Al, Cu, Au).

The LSAW characteristics were calculated by the finite element method using COMSOL Multiphysics software 5.6. A harmonic analysis was conducted with 1 V loaded on one IDT and 0 V on another. The cross-sectional schematic of the IDT/42LT/PML structure is shown in Figure 1a. The thickness of each layer is denoted as *d_e_*, *d_s_* and *d_PML_*. Only a period is considered, and the symmetric periodic boundary condition is applied to the field variables on the left (Γ*_L_*) and right surfaces (Γ*_R_*). A perfectly matched layer (PML) is placed at the bottom to decrease the model size and avoid wave reflection from the bottom.

Figure 1a also presents the typical displacement of an LSAW. The energy of the LSAWs is basically concentrated in the electrode layer under suitable conditions. Thus, the influence of the IDT thickness (*d_e_*) and the materials (Al, Cu, Au) on the LSAW’s performance is assessed by a quasi-3D FEM analysis, where the wavelength is *λ* = 1.5 µm, the piezoelectric layer thickness is *d_s_* = 7*λ* and the PML thickness *d_PML_* = *λ*.

The performance parameters of the LSAW, including the phase velocity (*V_p_*), electromechanical coupling coefficient (*K*^2^) and temperature coefficient of frequency (*TCF_r_*), are calculated when the thickness of three kinds of electrode materials, Al, Cu and Au, changes from 0.01 λ to 0.15 λ. *V_p_* determines the operation frequency and size of the SAW devices, which can be calculated from Equation (1), where *f_r_* is the resonance frequency. *K*^2^ determines the excited LSAW energy and bandwidth of the SAW devices, which can be calculated from Equation (2), where *f_a_* is the anti-resonance frequency. *TCF_r_* determines the influence of temperature on the resonance frequency of the SAW devices, which can be calculated from Equation (3), where *f_r_*|T = 25 °C and *f_r_*|T = 30 °C are the resonance frequencies at 25 °C and 30 °C, respectively [12]. The material constants of Al, Cu, Au and 42LT are taken from [13] and [14]. In the FEM simulation, we replaced different material constants at T = 25 °C and 30 °C to calculate the resonance frequencies, *f_r_*|T = 25 °C and *f_r_*|T = 30 °C, and then substituted them into Equation (3). In Equation (3), the thermal expansion coefficient of 42LT in the direction of propagation is α = 16.1 × 10^−6^/°C.

(1) $$V_{p}=f_{r}\times \lambda$$

(2) $$K^{2}=\frac{\pi f_{r}/2f_{a}}{\tan(\pi f_{r}/2f_{a})}$$

(3) $$TCF_{r}=\frac{f_{r}|T=30\;{}^{\circ}C-f_{r}|T=25\;{}^{\circ}C}{5f_{r}|T=25\;{}^{\circ}C}-\alpha$$

Figure 1b shows the *V_p_* of the LSAWs as a function of *d_e_* with different metal materials (Al, Cu, Au). The LSAW velocity decreases with the increase in the thickness of the electrode, but the falling range reduces from Au to Al. This is due to the density of the three electrode materials following the relationship: ρ_Au_ > ρ_Cu_ > ρ_Al_. It seems that Al is more preferable as an electrode material for obtaining a large wavelength for the LSAWs on 42LT substrates when operating at a high frequency. This is because a large wavelength means a wide IDT finger, which reduces lithographic difficulty.

Figure 1c shows the *K^2^* of the LSAWs as a function of *d_e_* with different metal materials (Al, Cu, Au). *K^2^* increases first and then decreases generally with the increase in the thickness of the three different electrodes from 0.01 λ to 0.15 λ. They reach a maximum value of 10.15% when *d_e_*_Al is 0.1 λ, 10.69% when *d_e_*_Cu is 0.05 λ and 10.66% when *d_e_*_Au is 0.02 λ, respectively. It is worth noticing that there are spikes when *d_e_*_Cu is 0.13λ and *d_e_*_Au is 0.07 λ, respectively. This may be due to the fact that the Rayleigh SAWs and LSAWs are slightly coupled in this situation.

Figure 1d shows the *TCF_r_* of the LSAWs as a function of *d_e_* with different metal materials (Al, Cu, Au). *TCF_r_* increases first and then decreases generally with the increase in the thickness of three different electrodes from 0.01 λ to 0.15 λ. It remains around −31 ppm/°C when the thickness of Al is no more than 0.1 λ, the thickness of Cu is no more than 0.06λ and the thickness of Au is no more than 0.04 λ, respectively. When the electrode thickness exceeds these limits, the temperature characteristics of the LSAWs become rapidly worse.

To sum up, the performance of the LSAWs on 42LT substrates is greatly affected by the electrode thickness and material. Similar to the Al electrode, the Cu and Au electrodes can achieve a high-performance LSAWs under some suitable conditions. But the LSAW’s performance with Cu and Au is more sensitive to the metal thickness, which means that less metal thickness error is permitted during the manufacturing process. Further, the Al electrode has the advantages of high velocity and low cost. Thus, this work employs Al as the metal material of the LSAWs on the 42LT substrate in the following sections. To achieve a large *K*^2^, the Al thickness is around 0.1 λ.

## 3. Transverse Leakage Suppression

**Figure 2:**
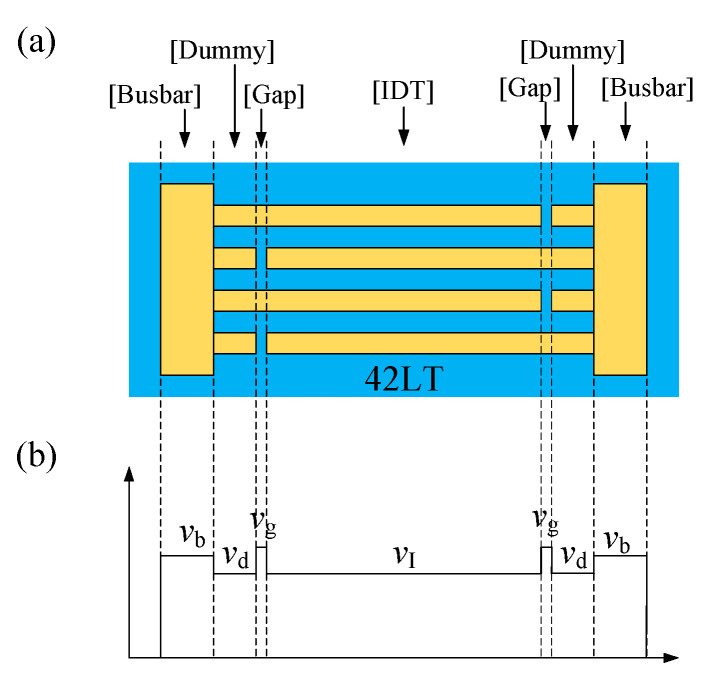
(**a**) Top view and (**b**) outline of SAW velocity for the conventional SAW resonator structure.

As described in Ref. [9], the velocity differences between the IDT and busbar regions lead to the transverse leakage of the LSAWs into the busbar region on the 42LT substrate. Figure 2 shows the schematic view of a conventional SAW resonator structure and the outline of the SAW velocity in each region. The SAW velocity of the IDT, gap, dummy and busbar regions is represented by *v*_I_, *v*_g_, *v*_d_ and *v*_b_, respectively. The following relationship holds: *v*_g_ > *v*_b_ > *v*_I_ = *v*_d_. As the slowness curve of the LSAWs on the 42LT substrate is concave [15], the transverse energy penetrates into the gap, dummy and busbar regions.

**Figure 3:**
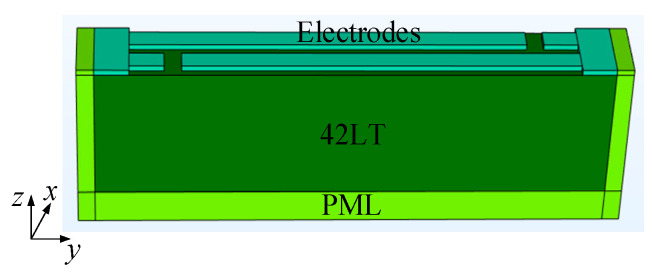
The 3D periodic FEM model.

In order to deeply investigate the effects of different gap and dummy lengths on the transverse leakage, we employed the 3D periodic finite element method (FEM) model built in COMSOL Multiphysics [16]. As shown in Figure 3, the layers are electrodes, the 42LT substrate, and the perfectly matched layer (PML) from the top to the bottom, respectively. The PML is placed around the model to avoid reflections from the boundaries. Only a period was considered, and the field variables at the left and right surfaces were set as periodic. Similar to the top view in Figure 2, the electrode layer can be divided into four regions (IDT, gap, dummy and busbar) along the transverse (y) direction. A harmonic analysis was conducted with 1 V loaded on one side of the IDT, dummy and busbar regions and 0 V on the other side.

The structural parameters used in the simulation are listed in Table 1, where the electrode material and thickness are determined according to the former section. In the fabrication process, there is usually an intermediate metal layer (titanium) used to improve the adhesion of aluminum to the substrate. Here, the electrode thickness includes 160 nm of aluminum and 2.6 nm of titanium, which is equal in mass to 164.3 nm of aluminum. The aperture length is close to the actual fabrication length in Section 4. With these parameters, the SAW resonances operate at a frequency of 2300 MHz~2600 MHz, which coincides with the band 40/41 SAW filter’s frequency.

**Figure 4:**
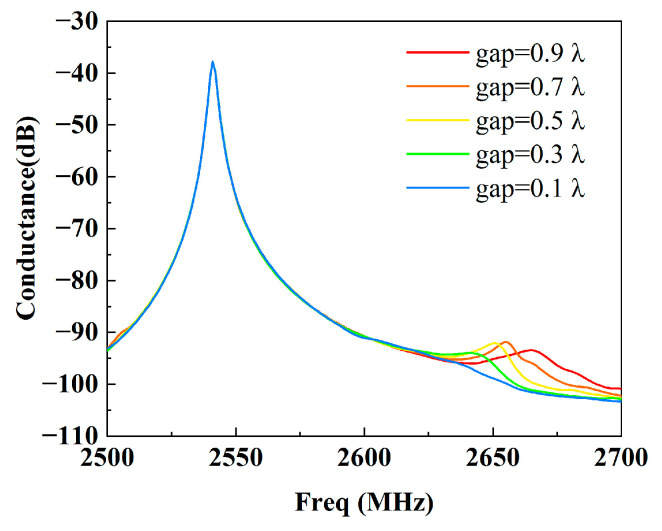
Calculated conductance for the different gap lengths without a dummy.

**Figure 5:**
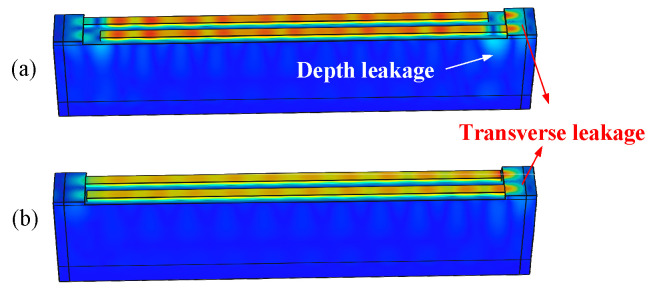
Displacement distributions at *f* = 2650 MHz for different gap lengths: (**a**) gap = 2 λ and (**b**) gap = 0.5 λ.

Firstly, we calculated the admittance dependence on the gap length without the dummy fingers from 2500 MHz to 2700 MHz with a step size of 1 MHz. Figure 4 shows the calculated conductance (the real part of the admittance) for different gap lengths from 0.1 λ to 0.9 λ with a step size of 0.2λ. The curves show that without a dummy, there are some bumps in the vicinity of *f* = 2650 MHz. When the gap is short, the bump is weak. Subsequently, we drew snapshots of the displacement distribution at *f* = 2650 MHz for the different gap lengths, as shown in Figure 5. It can be inferred that the bumps arise from the depth leakage and transverse leakage. A long gap leads to depth leakage at the end of the finger, which is due to a boundary discontinuity. Although a short gap can weaken the depth leakage, transverse leakage still occurs. This is due to the SAW velocity in the busbar region being faster than that in the IDT region.

Therefore, dummies are often employed as transverse reflectors to prevent SAW transverse leakage into the busbar region. To investigate the effects of a transverse reflector, we also calculated the admittance dependence on different dummy lengths. It should be noted that in this calculation, the gap length is 0.2 λ because this gap length approximates our lithographic limitation, though a shorter length achieves a higher performance.

**Figure 6:**
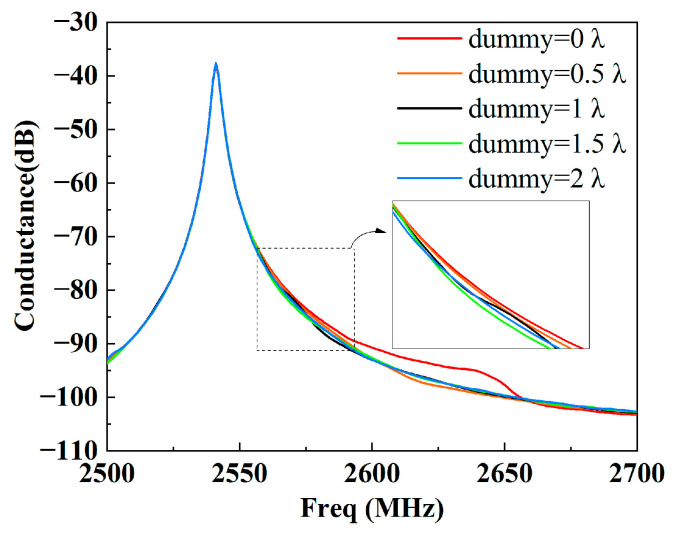
Calculated conductance at different dummy lengths.

**Figure 7:**
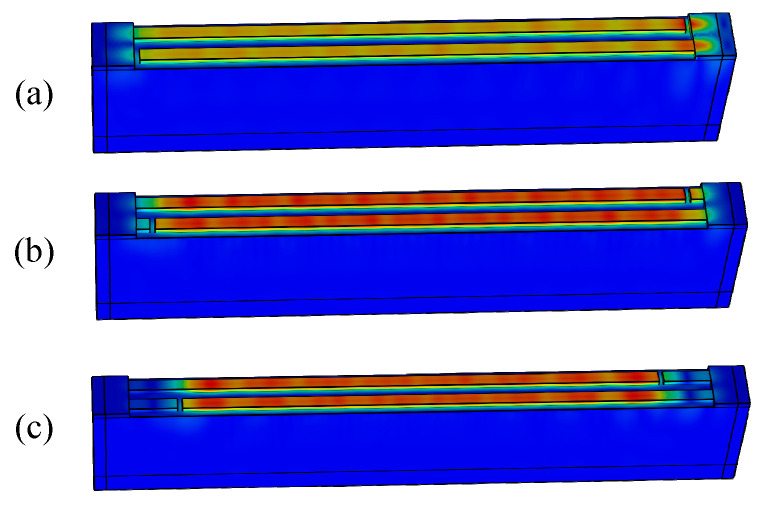
Displacement distributions with different dummy lengths: (**a**) dummy = 0, (**b**) dummy = 0.5 λ and (**c**) dummy = 2 λ.

Figure 6 shows the calculated conductance at different dummy lengths from 0 to 2 λ with a step size of 0.5 λ. It can be seen that with the dummy, the bumps at a high frequency (2650 MHz) are effectively suppressed, but those at a low frequency (2570 MHz) still exist. For a clear view, snapshots of the displacement distribution at *f* = 2570 MHz for the different dummy lengths were drawn. As depicted in Figure 7, the bright spots that appear in the busbar region will reduce when the dummy is employed. More less-bright spots appear in the busbar region of the long dummy structure (dummy = 2 λ) compared with the short one (dummy = 0.5 λ), which means that the long dummy is more effective in preventing the SAWs from penetrating into the busbar region than the short one. However, transverse leakage still slightly occurs with the long dummy, which results from the SAW velocity in the dummy region being identical to that in the IDT region.

**Figure 8:**
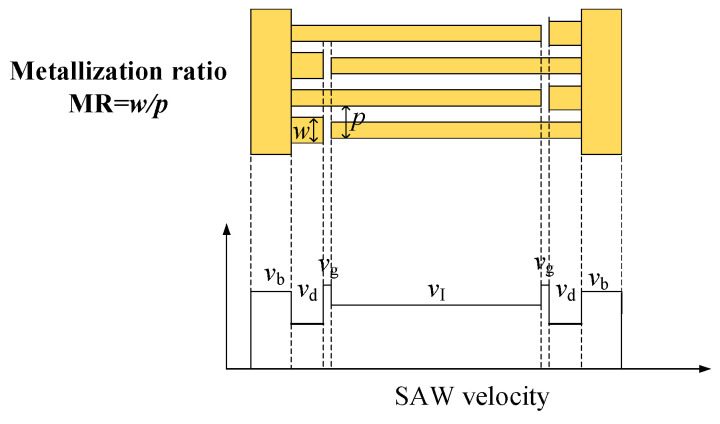
SAW velocity outline for the wider dummy technique.

To further suppress the transverse leakage, we propose a wider dummy technique, which makes the SAW velocity in the dummy region slower than that in the IDT region, as shown in Figure 8. In this situation, wider dummies serve as an energy barrier which can guide the SAWs in the transverse direction. To validate this, we calculated the admittances with different dummy metallization ratios (MRs) while the dummy length was 2 λ. The MR represents the ratio of the dummy width to the pitch, where the pitch is half of the wavelength (λ).

**Figure 9:**
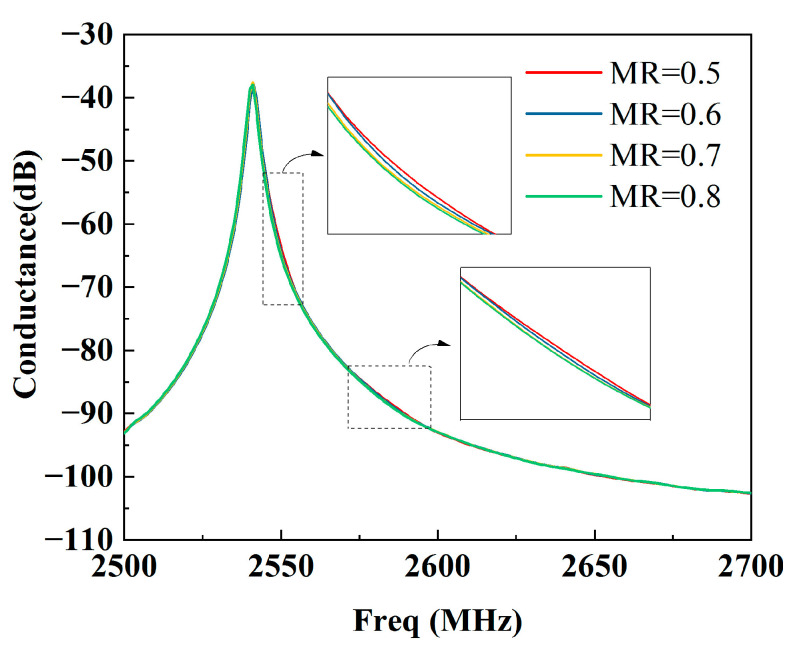
Calculated conductance at different MRs.

**Figure 10:**
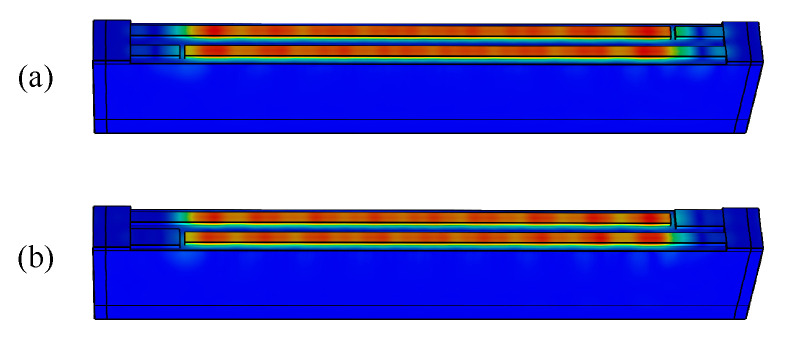
Displacement distributions with different dummy MRs: (**a**) MR = 0.5 and (**b**) MR = 0.8.

Figure 9 shows the calculated results. It can be seen that the bumps in the vicinity of *f* = 2550 MHz and 2575 MHz gradually fade away as the MR of the dummy increases from 0.5 to 0.8 with a step of 0.1. The displacement distributions of a normal dummy (MR = 0.5) and a wider dummy (MR = 0.8) are presented in Figure 10a,b, respectively. Obviously, it is too hard to see any bright spots in the wider dummy structure. Namely, the SAW energy is confined in the IDT region by the wider dummy structure. But they penetrate into the busbar region with the normal dummy structure. That means the wider dummy structure can suppress the transverse leakage more effectively.

To further evaluate the SAW energy confinement, we calculated the Bode_Q [17] of the three different structures, namely without a dummy (dummy = 0), with a normal dummy structure (dummy = 2 λ, MR = 0.5), and with a wider dummy structure (dummy = 2 λ, MR = 0.8). The formula is given as follows:

(4) $$Q=\frac{\omega *|S11|*group\_delay(S11)}{1-|S11{|}^{2}}$$

where *ω* = 2π*f*, and *S*11 is the return loss. The group delay of *S*11 is given by

(5) $$group\_delay(S11)=\frac{d(phase(S11))}{d\omega }$$

**Figure 11:**
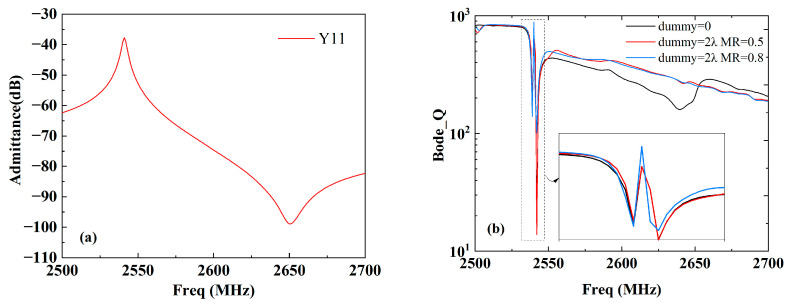
(**a**) Calculated admittance, Y11, and (**b**) Bode_Q of the three different structures.

The calculated results are shown in Figure 11, where Figure 11a shows the calculated admittance, Y11, for a clear view of the *f*r–*f*a band. Figure 11b shows the calculated Bode_Q from 2500 MHz to 2700 MHz. It can be easily seen that the difference in Bode_Q between *f*r (the resonance frequency) and *f*a (the anti-resonance frequency) is significantly enhanced with the normal dummy structure compared with the without-dummy structure. Moreover, the Bode_Q around *f*r is additionally enhanced by the wider dummy structure owing to the suppression of the transverse leakage. Interestingly, the Bode_Q values are almost identical below *f*r with the three different structures but much higher above *f*a with the without-dummy structure. This may be due to the end effects between the dummy fingers and the active IDT fingers.

## 4. Application to Band 40/41 SAW Filters

**Figure 12:**
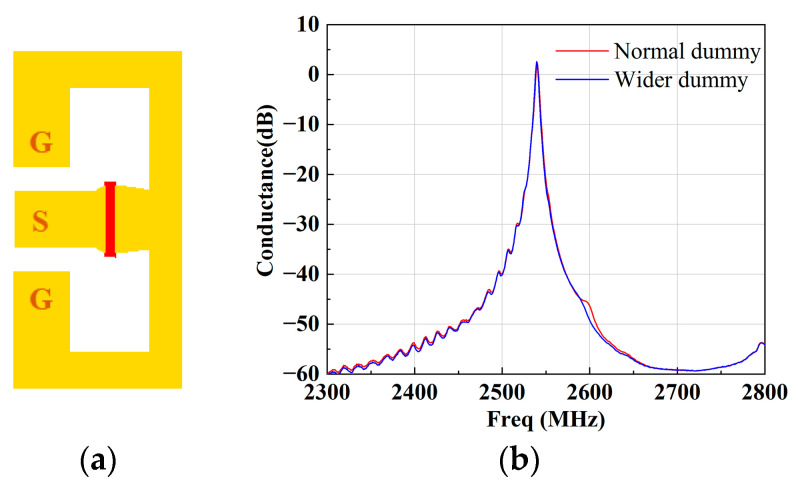
(**a**) Measured pattern and (**b**) conductance with different dummy structures.

We then extended the wider dummy technique to apply it to band 40 and 41 SAW filters. At first, an SAW resonator with an Al thickness of 164.3 nm and λ varying from 1.38 μm to 1.74 μm was experimentally fabricated to verify the simulated results. The resonators contained 75 pairs of IDT fingers and 10 pairs of reflector fingers, and the aperture was 20 λ. The resonators were measured via a one-port ground–signal–ground (GSG) probe measurement, as shown in Figure 12a. One of the measured results is shown in Figure 11b, where λ is 1.6 μm and the corresponding resonance frequency is 2537 MHz. It can be easily seen from Figure 12b that bumps emerge in the vicinity of *f* = 2550 MHz and 2600 MHz with the normal dummy structure (MR = 0.5). Predictably, the bumps fade away with the wider dummy structure (MR = 0.6). This measured results agree well with the simulated ones.

**Figure 13:**
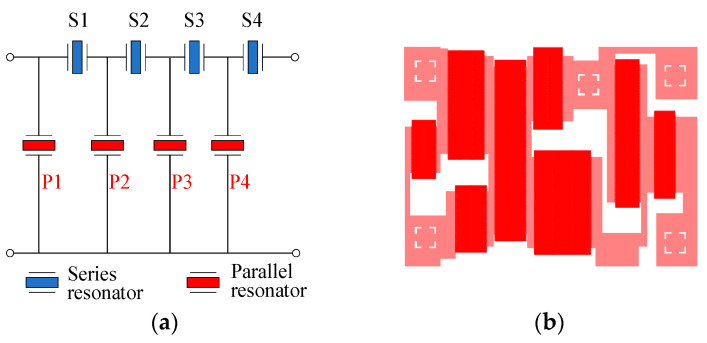
(**a**) Circuit topology and (**b**) layout of the B40 SAW filter.

**Figure 14:**
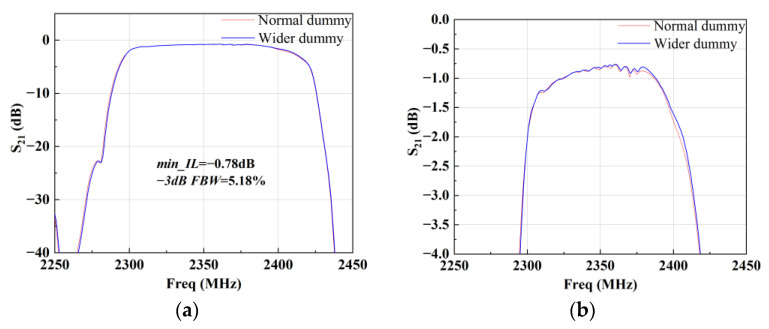
Measured S21 of the B40 SAW filter with different dummy structures: (**a**) overall view and (**b**) zoomed-in view.

Then, B40 and B41 SAW filters were designed and fabricated. They were designed using the coupling-of-modes (COM) theory [18]. Figure 13 shows the circuit topology and layout of the B40 SAW filter. The filter is composed of four series and four parallel SAW resonators to achieve an out-of-band rejection of −30 dB. Figure 14 shows the corresponding measured insertion loss, S21, with different dummy structures in an overall and zoomed-in view. With this topology, the B40 SAW filter achieves a center frequency of 2356 MHz, a minimum insert loss of −0.78 dB, and a −3 dB fractional bandwidth (FBW) of 5.18%. The out-of-band rejection is about −25 dB. As depicted in Figure 14b, with the wider dummy structure, the insertion loss of more than 0.2 dB is improved around the higher edge, and the bandwidth of −2 dB is improved from 104 MHz to 106.4 MHz. This means that more energy has been confined in the active region by the wider dummy structure.

**Figure 15:**
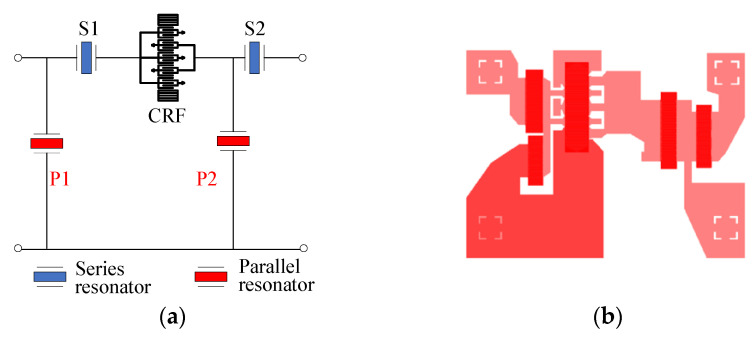
(**a**) Circuit topology and **(b**) layout of the B41 SAW filter.

**Figure 16:**
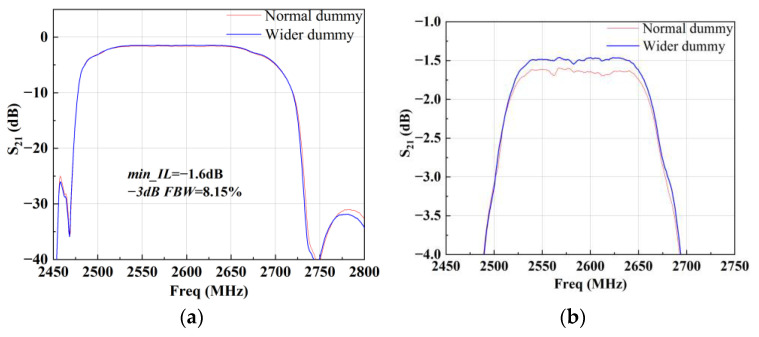
Measured S21 of the B41 SAW filter with different dummy structures: (**a**) overall view and (**b**) zoomed-in view.

Figure 15 shows the circuit topology and layout of the B41 SAW filter. The topology is composed of a longitudinally coupled resonator filter (CRF) [19] and two series and two parallel SAW resonators. The CRF is employed to achieve a high out-of-band rejection, while the ladder resonators are employed to obtain a steep roll-off characteristic. Figure 16 shows the corresponding measured insertion loss, S21, with different dummy structures in an overall and zoomed-in view. With this topology, the B41 SAW filter achieves a center frequency of 2592 MHz, a minimum insert loss of −1.6 dB, and a −3 dB fractional bandwidth (FBW) of 8.15%. The out-of-band rejection is about −30 dB. It can be found that the insert loss and 3 dB FBW of the B41 SAW filter are both larger than those of the B40 SAW filter. This is because the CRF couples high-order longitudinal modes, which can help achieve a wider bandwidth at the cost of insert loss. An FBW of 8.15% is obtained by external matching. As depicted in Figure 16b, the insertion loss of the whole passband is improved by more than 0.2 dB, and the bandwidth of −2 dB is improved from 144.75 MHz to 148.5 MHz with the wider dummy structure.

Essentially, both of the improvements in the performance of the band 40 and 41 SAW filters are owing to the suppression of transverse leakage by the wider dummy structure. And this structure does not require any additional lithographic or depositing processes.

**Figure 17:**
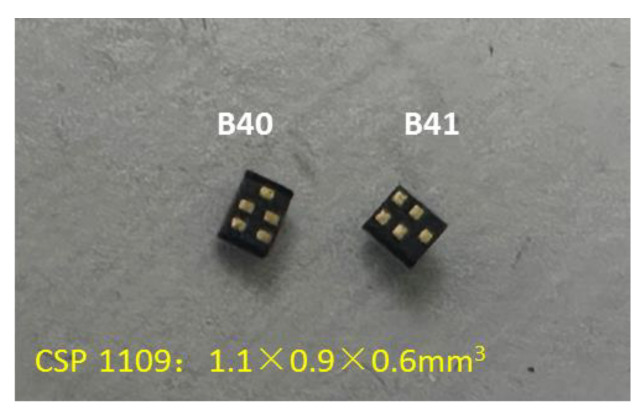
Packaged B40 and B41 SAW filters.

**Figure 18:**
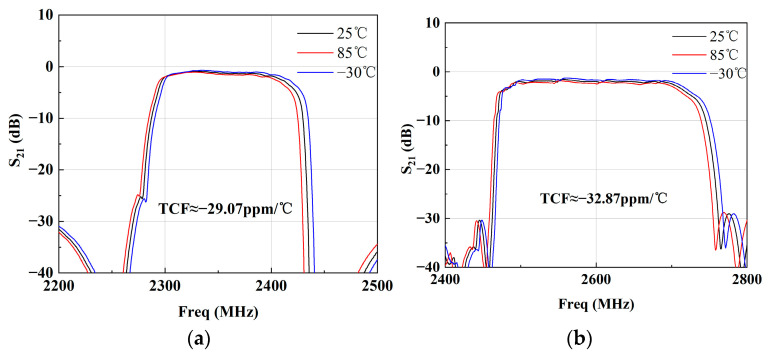
Measured S21 of (**a**) B40 SAW filter and (**b**) B41 SAW filter at three different temperatures: −30 °C, 25 °C and 85 °C.

In the end, the band 40 and 41 SAW filters were packaged in the Chip Scale Package 1109 (CSP 1109) [20], as shown in Figure 17. This means that the sizes of the SAW filters are both 1.1 × 0.9 × 0.6 mm^3^. After packaging, the temperature characteristics of the band 40 and 41 SAW filters were measured at three different temperatures: −30 °C, 25 °C and 85 °C. Figure 18 shows the measured S21 of the band 40 and 41 SAW filters at the different temperatures. When the temperature rises from −30 °C to 85 °C, the frequency moves down; meanwhile, the insert loss increases. When the temperature falls from 25 °C to −30 °C, the insert loss of the band 40 SAW filter decreases by 0.18 dB, and the frequency moves up from 2356 MHz to 2359.75 MHz. The insert loss of the band 41 SAW filter decreases by 0.27 dB, and the frequency moves up from 2592 MHz to 2596.68 MHz. When the temperature rises from 25 °C to 85 °C, the insert loss of the band 40 SAW filter increases by 0.19 dB, and the frequency moves down from 2356 MHz to 2351.9 MHz. The insert loss of the band 41 SAW filter increases by 0.29 dB, and the frequency moves down from 2592 MHz to 2586.88 MHz. The measured TCFs of the band 40 and 41 SAW filters are about −29.07 ppm/°C and −32.87 ppm/°C, respectively. They are very close to the simulated ones (about −31 ppm/°C at *de*_Al = 0.1λ). The slight difference may be due to errors in the material constants, and the actual *de*_Al is not strictly 0.1λ for different SAW resonator elements.

## 5. Conclusions

This paper presents B40 and B41 SAW filters on a 42LT substrate with the suppression of transverse leakage. Firstly, the LSAW characteristics (*V_p_*, *K*^2^ and *TCF_r_*) with different metal materials (Al, Cu, Au) and thicknesses (0.01 λ~0.15 λ) were calculated by the FEM. It showed that the performance of LSAWs on the 42LT substrate was greatly affected by the electrode thickness and material. Similar to the Al electrode, the Cu and Au electrodes can achieve high-performance LSAWs under some suitable conditions. But the LSAW performance with Cu and Au is more sensitive to the metal thickness, which means that less metal thickness error is permitted during the manufacturing process. Thus, to achieve a large *K*^2^, a thickness of 0.1λ for Al was employed as the IDT of the B40 and B41 SAW filters on the 42LT substrate. Then, to deeply understand the transverse leakage and suppress it, the effects of different gap and dummy lengths on the leakage were simulated through a 3D FEM model. The simulated results indicated that a short gap and long dummy could partly suppress the depth and transverse leakages, respectively. Further, with the wider dummy structure, the transverse leakage was almost completely suppressed, and Bode_Q was significantly enhanced around the resonance frequency. Finally, the wider dummy technique was extended to be applied to band 40 and 41 SAW filters. The filters were designed according to COM theory and packaged in CSP 1109. The band 40 SAW filter achieved a center frequency of 2356 MHz, a minimum insert loss of −0.78 dB, a −3 dB FBW of 5.18% and a TCF value of −29.07 ppm/°C. The band 41 SAW filter achieved a center frequency of 2592 MHz, a minimum insert loss of −1.6 dB, a −3 dB FBW of 8.15% and a TCF value of −32.87 ppm/°C. The measured results showed that with the wider dummy structure, the band 40 and 41 SAW filters achieved a more than 0.2 dB improvement in the insertion loss, a wider bandwidth and a steeper roll-off characteristic. This technique may also be extended to other-band SAW filter applications.

## Figures and Tables

**Table 1:** Structural parameters.

Simulation Parameters	
Substrate material	42LT
Electrode material	Aluminum
Wavelength (λ)	1.6 μm
Electrode thickness (de)	164.3 nm
Aperture length (W)	30 μm

## Data Availability

The authors have access to all the data in the study (for original research articles) and accept responsibility for their validity.

## References

[1] Zhang M., Luo G., Feng L., Kui L. A novel dual-band planar monopole mobile phone antenna for TD-LTE and TD-SCDMA applications. Proceedings of the 2017 4th International Conference on Systems and Informatics (ICSAI).

[2] Vadlamudi R., Kumar D.S., Triple-Band D.P. Low Profile and High Gain Antenna with High Isolation for 4G (Band 40/41) and 5G BTS Applications. Proceedings of the 2020 IEEE 3rd 5G World Forum (5GWF).

[3] Iwamoto H., Takai T., Takamine Y., Nakao T., Fuyutsume T., Koshino M. Transverse Modes in I.H.P. SAW Resonator and Their Suppression Method. Proceedings of the 2018 IEEE International Ultrasonics Symposium (IUS).

[4] Inoue S., Solal M. LT/Quartz Layered SAW Substrate with Suppressed Transverse Mode Generation. Proceedings of the 2020 IEEE International Ultrasonics Symposium (IUS).

[5] Koskela J., Knuuttila J.V., Makkonen T., Plessky V.P., Salomaa M.M. Acoustic loss mechanisms in leaky SAW resonators on lithium tantalate. Proceedings of the 2000 IEEE Ultrasonics Symposium. Proceedings.

[6] Solal M., Gratier J., Gamble K., Abbott B., Kook T., Chen A. Transverse modes suppression and loss reduction for buried electrodes SAW devices. Proceedings of the 2010 IEEE International Ultrasonics Symposium.

[7] Solal M., Chen L., Gratier J. Measurement and FEM/BEM simulation of transverse effects in SAW resonators in lithium tantalate. Proceedings of the 2010 IEEE International Ultrasonics Symposium.

[8] Tsutsumi J., Inoue S., Iwamoto Y. Extremely low-loss SAW filter and its application to antenna duplexer for the 1.9 GHz PCS full-band. Proceedings of the IEEE International Frequency Control Symposium and PDA Exhibition Jointly with the 17th European Frequency and Time Forum, 2003. Proceedings of the 2003.

[9] Inoue S., Tsutsumi J., Matsuda T., Ueda M., Ikata O., Satoh Y. (2007). Analysis and Suppression of Side Radiation in Leaky SAW Resonators. Proc. IEEE Trans. Ultrason. Ferroelectr. Freq. Control.

[10] Nakamura H., Komatsu T., Nakanishi H., Tsurunari T., Fujiwara J. Reduction of transverse leakage for SAW resonators on LiTaO_3_ substrate. Proceedings of the 2012 IEEE International Ultrasonics Symposium.

[11] Solal M., Gratier J., Kook T. (2009). A SAW resonator with two-dimensional reflectors. Proceedings of the Frequency Control Symposium, 2009 Joint with the 22nd European Frequency and Time Forum.

[12] Zhang S.K., Wang H.L., Zhang P., Cao G. (2023). High-Performance SAW Resonator with Spurious Mode Suppression using Double-layer Electrode Transverse Modulation. Semicond. Sci. Technol..

[13] Ballandras S., Gavignet E., Bigler E., Henry E. (1997). Temperature derivatives of the fundamental elastic constants of isotropic materials. Appl. Phys. Lett..

[14] Kovacs G., Anhorn M., Engan H.E., Visintini G., Ruppel C.C.W. Improved material constants for LiNbO_3_ and LiTaO_3_. Proceedings of the 1990 Ultrasonics Symposium.

[15] Xiao Q., Ji X., Ma X., Cai P. (2020). A New General Form of 2-D Coupling-of-Modes Equations for Analysis of Waveguiding in Surface Acoustic Wave Devices. IEEE Trans. Ultrason. Ferroelectr. Freq. Control.

[16] Xiao Q., Ma X., Wang W., Fan Y., Cai P., Ji X. (2021). High-Performance SAWR Strain Sensor With Piston-Mode Operation. IEEE Sens. J..

[17] Feld D.A., Parker R., Ruby R., Bradley P., Dong S. After 60 years: A new formula for computing quality factor is warranted. Proceedings of the 2008 IEEE Ultrasonics Symposium.

[18] Plessky V., Koskela J. (2000). Coupling-of-modes Analysis of SAW Devices. Int. J. High Speed Electron. Syst..

[19] Meltaus J., Plessky V.P., Harma S., Salomaa M.M. (2005). Low-loss, multimode 5-IDT SAW filter. IEEE Trans. Ultrason. Ferroelectr. Freq. Control.

[20] Goetz M., Jones C. Chip scale packaging techniques for RF SAW devices. Proceedings of the 27th Annual IEEE/SEMI International Electronics Manufacturing Technology Symposium.

